# New, Improved Treatments for Chagas Disease: From the R&D Pipeline to the Patients

**DOI:** 10.1371/journal.pntd.0000484

**Published:** 2009-07-07

**Authors:** Isabela Ribeiro, Ann-Marie Sevcsik, Fabiana Alves, Graciela Diap, Robert Don, Michael O. Harhay, Shing Chang, Bernard Pecoul

**Affiliations:** 1 Drugs for Neglected Diseases *initiative*, Geneva, Switzerland; 2 Masters of Public Health Program, University of Pennsylvania School of Medicine, Philadelphia, Pennsylvania, United States of America; Universidad de Buenos Aires, Argentina

## Introduction

Endemic throughout Latin America with a prevalence rate of approximately 1.4%, Chagas disease (CD) is estimated to kill 14,000 people every year, which is more people in the region each year than any other parasite-born disease, including malaria [1],[2]. Brazilian physician Carlos Chagas first described CD exactly a century ago [3], and its socioeconomic impact makes it the most important parasitic disease in the Americas [4]. Estimated to infect somewhere between 8 to 14 million people, CD both afflicts the poor and, like other neglected tropical diseases, “promotes poverty” [2],[5]. Through its impact on worker productivity, and by causing premature disability and death, CD annually costs an estimated 667,000 disability-adjusted life years lost [1],[6]. In the case of Brazil alone, losses of over US$1.3 billion in wages and industrial productivity were due to the disabilities of workers with CD [7].

CD is an important public health issue, both in Latin America and increasingly around the world: the infection rate in endemic areas is estimated to be 1.4% [8], with geographic variation from 0.1% to 45.2% [9]. Vectorial transmission has been significantly reduced due to control efforts like the Southern Cone Initiative [10],[11] and others [11],[12]. However, there are areas producing new cases such as regions untouched by vector control efforts [13], special areas with non-domiciliated triatomine [14], and the Amazon region with recent cases reported via oral transmission and by wild triatomine [15]. And still to this day, millions of patients remain without adequate treatment for this silently debilitating and potentially fatal disease. Although no official global figures exist, it is estimated that no more than 1% of those infected are believed to receive any treatment at all. An increasing number of CD patients are also seen in non-endemic, developed countries because of globalization and the movement of unknowingly infected people from Latin America to other parts of the world [16],[17],[18]. The appearance of *Trypanosoma cruzi* in blood banks in the United States has led the Food and Drug Administration (FDA) to recently issue a draft guidance on CD screening [19].

## The Need for New, Improved Treatments

To better understand the need for new treatments, it is important to review a bit of CD pathology and clinical evolution. Caused by infection with the protozoan parasite *T. cruzi*, CD starts with an acute phase in which the parasitemia is often high and parasitological diagnosis can be made by direct microscopic examination of fresh blood. This disease phase (in which 2%–8% of children die) [20],[21] frequently passes undiagnosed in the absence of active screening programs, as CD manifests itself with a febrile and toxemic illness having non-specific symptoms reminiscent of any childhood infection. If untreated, the disease transitions into a clinically silent, indeterminate chronic phase. Later, 10 to 30 years after the initial infection, approximately 30% of infected people will experience the symptomatic, chronic stage characterized by severe organ pathologies primarily involving the cardiac and gastrointestinal systems [22],[23]. During the long-lasting chronic phase, parasites are primarily in the tissues, thereby rendering direct parasitological diagnosis difficult or impossible. At this stage, diagnosis is based on serology and more recently on molecular diagnosis via polymerase chain reaction (PCR). Hemoculture and xenodiagnosis can also be done, but with limited sensitivity.

**Figure 1:**
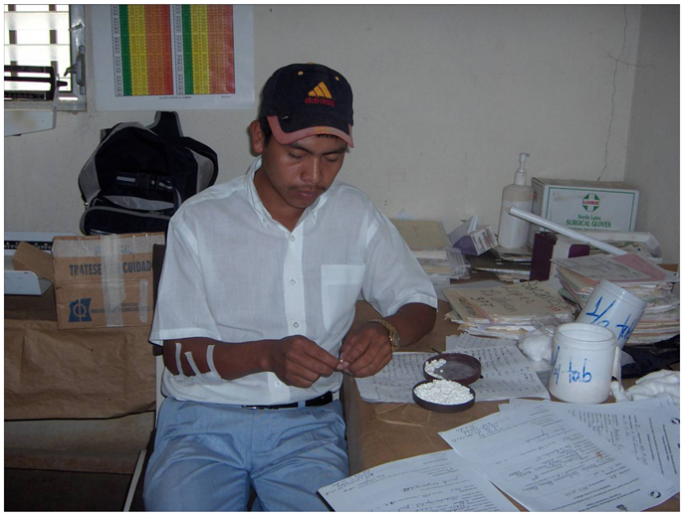
Fractionation of benznidazole tablets. At a health post in Honduras, benznidazole tablets are fractionated by hand into ½ and ¼ tablets. Fractionation of tablets is not ideal, as there is a high risk of delivering the improper dosage, thereby raising concerns about safety, efficacy, and decreased stability. (Photo courtesy of the National Chagas and Leishmaniasis Control Program of Honduras.)

Current treatment is limited to only two drugs: nifurtimox (Lampit; Bayer) and benznidazole (LAFEPE-BENZNIDAZOLE, Laboratorio Farmacêutico do Estado de Pernambuco [LAFEPE]). Unfortunately, these drugs are limited to the treatment of children with acute infection and early chronic disease (<12 years old) [24], with growing evidence for treatment in indeterminate disease [25],[26],[27]. For the chronic phase with target organ involvement, few studies support their use as parasitological therapy [27],[28], but the BENEFIT trial supported by the Special Programme for Research and Training in Tropical Diseases (TDR) is expected to fill this knowledge gap [29]. Even in children, who are known to better tolerate treatment with these nitroheterocyclic compounds than adults, the cure rate for chronic indeterminate cases is up to 62% at 2 year follow-up [24],[25],[30], and it may vary according to population and geographical location [25],[26],[27],[31]. Both drugs require a 30–60 day treatment period that fosters a high rate of patient non-compliance, and dose- and time-dependent toxicity is also seen [32]. No pediatric strength or formulation is available for benznidazole (Figure 1); a 30-mg tablet strength of nifurtimox was developed and registered, but is not currently available. Because of the poverty and remoteness of the primary target population, guaranteeing access to diagnosis and treatment is a challenge.

Although new, improved treatments against CD are urgently needed, no new anti-CD drugs are in clinical development, and only one class of drugs, the antifungal triazoles, have demonstrated potential for therapeutic switching. While some promising academic and non-commercial drug discovery efforts exist, the current drug research and development (R&D) pipeline is still very limited, with no new drug expected within the next 3–4 years.

## Barriers to Development and Evaluation of Treatments

A lot of research has been conducted on the parasite *T. cruzi* over the past century, culminating in the sequencing of its genome and proteome in 2005 [33],[34]. However, basic research on *T. cruzi* has yet to translate into new therapeutic tools for CD for a number of reasons.

In early stage research, many compounds might show promising activity against *T. cruzi*, but there is little standardization among the protocols or parasites used for each assay. Reproducibility has sometimes been difficult across laboratories; several so-called active compounds have been identified using assays not relevant to disease pathology (i.e., screening against parasitic epimastigotes and trypomastigotes) and many screening labs do not have (1) capacity/expertise to run assays with a reasonable throughput due to the nature of the *T. cruzi* assay, (2) pharmaceutical knowledge to conduct drug development on their hits, or (3) collaborations with partners having this knowledge (work stops after publication of results).

Few rigorous clinical trials have been conducted in CD [24],[26],[28]. For years, one of the important challenges in drug development for CD has been the evaluation of drug efficacy in the population representing the highest disease burden, patients with chronic indeterminate CD. Such patients do not present any clinical disease manifestation, and serological testing may remain positive for 5 years or even longer after treatment. To date, there are no randomized clinical trials evaluating the impact of treatment at the indeterminate phase of disease as it evolves into chronic cardiac or gastrointestinal disease. Since these manifestations occur in ∼30% of patients over 10–30 years after infection, such clinical trials would require very large sample sizes and decades of follow-up, and are therefore practically unfeasible. These concerns have contributed significantly to the paucity of new drugs that have been clinically assessed as CD treatments—clinical research is simply deemed “too difficult”. Hence, new research tools in designing clinical trials and surrogate markers of cure are needed.

## Responding to the Need—Promising Developments with New Partnerships

Difficult challenges lie ahead in the quest for the elimination of CD, as was acknowledged by the World Health Organization (WHO) in its recent report to the World Health Assembly [35], even as several new initiatives emerge on both the control and the research landscape.

One such development is the creation of non-profit product development partnerships (PDPs) working to fill the gaps in essential health tools for neglected diseases [36]. These emergent PDPs offer a valuable alternative model, as R&D is no longer financed by a product's sale price. In the case of CD, the Drugs for Neglected Diseases *initiative* (DNDi), a PDP, is currently working to build a well-balanced and robust CD-specific portfolio that urgently addresses the needs of CD patients. Improved treatments and research tools are required—DNDi aims to deliver an effective, non-toxic, inexpensive treatment proven effective for the acute, indeterminate, and chronic phases of CD. Work is also ongoing to develop a pediatric formulation of benznidazole, as this could represent a great improvement in point-of-care case management.

The changes seen in the past decade offer a new landscape in which to collaborate and to advance improved treatments for neglected diseases like CD, but, to ensure that these efforts are sustained and strengthened, greater investments (complemented with new and adapted funding mechanisms) are needed from both governments and the private sector. DNDi continues to identify and engage partners, so as to ensure that a well-balanced pipeline is established for CD, one of its three diseases of primary focus.

## Filling Gaps in the Pipeline

Matching needs and opportunities, DNDi's portfolio is a mix of projects in-sourced at any stage of the development process, from early discovery through post-registration, with the objective to bring new, field-relevant tools to patients in the shortest time and most efficient way possible. Preclinical and clinical development activities are streamlined and focused on the ultimate goal: a new treatment that reaches patients and contributes to improved disease control.

The CD-specific portfolio balances short- and long-term objectives. In the short and mid-term, the aim is for better use of existing treatments through new formulations, therapeutic switching, and combination therapy. In the long term, new chemical entities must be developed. Another important element in DNDi's strategy for CD is to address the methodological constraints that impact the design of clinical studies.

In order to best meet research opportunities and most immediately address patient needs, DNDi utilizes a target product profile (TPP). As a hypothetical “package insert”, the TPP contains elements that describe the ideal product to guide the development process. Table 1 gives an overview of the ideal and minimally acceptable TPP for chronic indeterminate CD.

**Table 1:** Target Product Profile for Developing a Treatment for Chronic Indeterminate Phase of CD.

	Acceptable	Ideal
**Target label**	Early chronic/indeterminate CD	Early chronic/indeterminate CD + Reactivations (Immunocompromised)
***T. cruzi*** ** sub-species**	TcI+TcII	TcI+TcII
**Distribution**	All areas	All areas
**Target population**	Immunocompetent	Immunocompetent + Immunocompromised
**Adult/children**	Adult	All
**Clinical efficacy**	Superiority over benznidazole in all endemic regions (parasitological)	70% (parasitological and serological)
		>95% cure for reactivated patients (parasitological and serological)
**Resistance**	Active against nitrofuran- and nitroimidazole-resistant *T. cruzi* strains	Active against nitrofuran- and nitroimidazole-resistant *T. cruzi* strains
**Safety**	Superiority to benznidazole	Superiority to benznidazole
	3 clinical evaluations plus 2 standard laboratory evaluations during treatment	No monitoring needed during treatment
**Contraindications**	Pregnancy/lactation	None
**Precautions**	No genotoxicity; no prolongation of QTc interval	No genotoxicity; no teratogenicity; no negative inotropic effect; no prolongation of QTc interval
**Interactions**	No clinically significant interaction with anti-hypertensive, anti-arrythmic, or anticoagulants drugs	None
**Presentation**	Oral	Oral
**Stability**	3 years, climatic zone IV	5 years, climatic zone IV
**Dosing regimen**	Comparable to systemic antifungal treatments	Two times a day for 60 days

**Figure 2:**
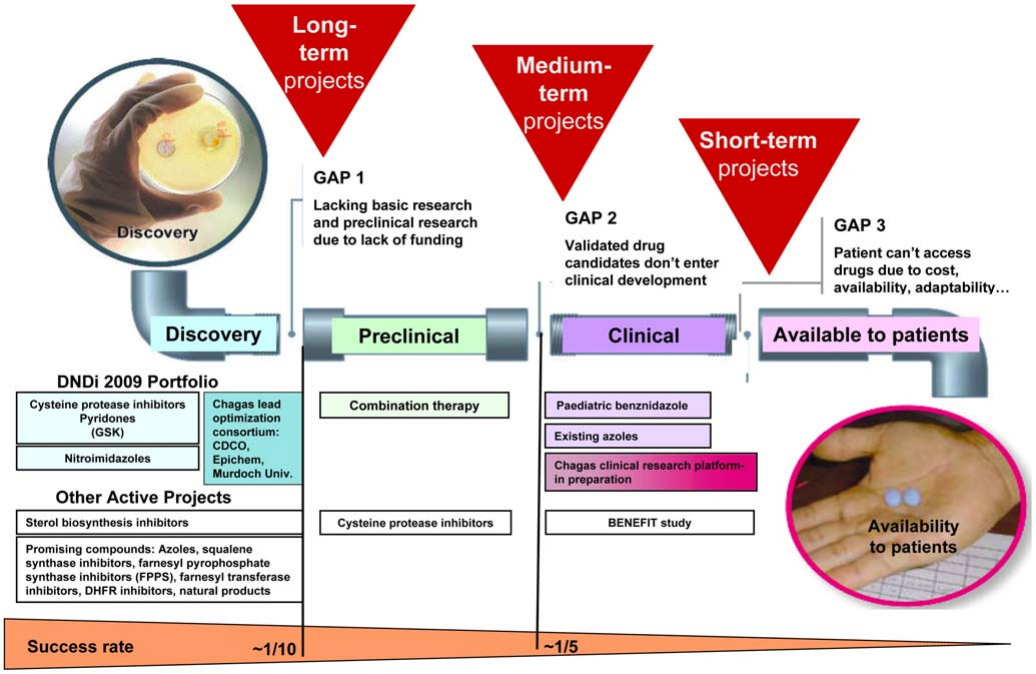
Ongoing drug R&D projects on Chagas disease. There are a few promising projects at early-stage discovery and clinical stages; however, the high attrition rate of the pipeline means that only one in ten compounds will be progressed from discovery into preclinical testing; and in late stages, only one in every five drugs that enter clinical trials becomes available to patients. Success rate based on estimates from Nwaka et al. 2003 [37].

A number of research activities hold promise at various stages throughout the pipeline, although it is clear that more research is needed (Figure 2). The high attrition rate of the pipeline is well known—even in late stages, only one in every five drugs that enter clinical trials becomes available to patients [37].

Highlighted below are some of DNDi's key activities along with some promising work being done elsewhere at institutions like Fiocruz, the University of California, San Francisco (UCSF), and the University of Washington. These activities are divided below by how long the development time will roughly take.

### Long-Term Projects (>6 Years)

#### Drug discovery

Some of the promising targets in *T. cruzi* include protein prenylation, hypoxanthine-guanine phosphoribosyltransferase, cysteine proteases [38],[39], topoisomerases [40], 14-demethylase inhibitors [41],[42], squalene synthase inhibitors [43], farnesyl pyrophosphate synthase inhibitors [44], farnesyl transferase inhibitors [45],[46], dihydrofolate reductase inhibitors [47], and natural products like canthinones, quinolines, naphthoquinones, and lignans [48],[49],[50].

Much of these data must still be confirmed by additional laboratories: key elements in DNDi's drug discovery process include (1) accessing broad chemical diversity through a number of different sources and partnerships such as a natural products screening network and collaborations with pharmaceutical companies, (2) ensuring standard operating procedures in place for in vitro and in vivo assays to ensure that screening at different sites and with different groups are comparable, and (3) increasing screening capacity for CD.

An important challenge for the screening of new compounds is the limited output of currently available screening methodologies. In a partnership at the forefront of technology development, DNDi and Institute Pasteur Korea are working to develop a visual-based high-throughput screening platform for *T. cruzi*. High-throughput screening offers the possibility of more rapid hit identification to be progressed as drug candidates.

#### Lead optimization (screening to drug candidate)

In 2008, a lead optimization consortium was set up by DNDi for CD so as to engage in a critical, iterative process that helps to optimize the efficacy of a lead compound while minimizing its toxicity. This consortium includes institutions in Australia (Monash and Murdoch Universities and Epichem) and Brazil (Universidade Federal de Ouro Preto) and consists of a group of analytical and medicinal chemists, pharmacologists, and parasitologists with rapid turnaround facilities or compound assessment.

### Medium-Term Projects (3–6 Years)

#### Therapeutic switching

With the high attrition of early screening and lead optimization efforts, a key approach in minimizing the risks and length of drug R&D time is to evaluate compounds registered or in clinical development for other indications with demonstrated in vitro and/or in vivo activity in CD. A potential target for therapeutic switching is ergosterol biosynthesis, a pathway effectively targeted for antifungal therapy that shares considerable similarity with the trypanosome pathway. However, most of the clinically employed sterol biosynthesis inhibitors (such as ketoconazole and itraconazole) are not able to induce complete parasitological cure in human Chagas disease and animal models [51].

A new generation of antifungal triazoles including posaconazole, voriconazole, and ravuconazole, show considerable promise as anti-trypanosomal agents. The marketed antifungal drug posaconazole (Noxafil, Schering-Plough) has previously been shown to induce parasitological cure in mice with acute and chronic infections, including benznidazole-resistant strains [48],[52]. It is considered the leading azole candidate for proof-of-concept evaluation. Two other triazole derivatives, ravuconazole (Eisai) and TAK-187 (Takeda), have shown encouraging in vitro and in vivo results [53],[54]. Both products have completed Phase I testing and are good candidates for further assessment as potential CD treatments.

#### Combination treatment

A main limitation to the broader use of etiological treatment in CD is the poor tolerability reported with currently available treatments. Side effects of benznidazole and nifurtimox are both time- and dose-dependent [48]. Combination therapy could improve treatment efficacy; could reduce dosage, treatment duration, and toxicity; and could also prevent the potential development of parasitic resistance to currently available treatments. Azole derivatives have shown synergistic anti–*T. cruzi* effects, in vitro and in vivo, with benznidazole and other compounds involved in the sterol biosynthesis pathway [42]. Taking these results into consideration, DNDi has begun preclinical studies with the objective of reducing the dose and duration of current CD treatments by systematically evaluating these two drugs in combination with azole compounds.

### Short-Term Projects (<3 Years)

#### Reformulation

Since the 1990s, there has been consensus for the early diagnosis and treatment of children and adolescents in the early indeterminate (chronic) phase of CD. Young children remain an important target population for treatment despite decreasing vectorial transmission, because congenital infection may remain an important mode of transmission for at least another generation. This is not reflected in the current treatment options, as current drugs are formulated as tablets for adults, that is, not adapted to children's weights. Tablet fractionation (Figure 1) and extemporaneous formulations are needed to treat most children; these procedures increase the likelihood of improper dosages and raise safety concerns, particularly in the very young and malnourished, as well as concerns about reduced efficacy (due to the addition of diluents) and stability.

A number of approaches have been examined to best meet the need of developing a new pediatric formulation that is affordable, age-adapted, and easy to comply with: an improved solution at the Universidad Nacional de Rosario Argentina and an adapted, dispersible tablet through a collaboration between LAFEPE and DNDi. Signed in July 2008, this collaboration seeks to develop and file for registration a dispersible pediatric tablet for the treatment of CD in endemic countries by the end of 2010.

### Clinical Research—Tackling the Challenges

Outside of specific drug development projects, DNDi is working to address a number of issues that could make clinical research “less difficult”: (1) ***Methodological issues for proof-of-concept evaluation in CD***—the long period for seroconversion after parasite elimination in CD presents an important challenge in the evaluation of etiological treatment. In recent years, an increasing body of data has pointed to a strong biological rationale for the use of parasitological outcomes as surrogate markers of therapeutic response in CD. A TDR-sponsored study for the standardization and validation of qualitative PCR testing for *T. cruzi* has just been completed, which represents a valuable first step for future clinical trials. Further work is still needed for validation of quantitative PCR and better definition of procedures for employment in drug studies. (2) ***Clinical site identification***—clinical trial sites must be identified that will ensure adequate recruitment of patients with different stages of the disease and who are infected with different strains of *T. cruzi*. (3) ***Clinical research strengthening platform for CD***—the Chagas Platform is being formed in 2009 with various partners to strengthen clinical research capacities by developing a critical mass of expertise, strengthening institutional research capacity, and supporting an environment conducive to quality research in order to review and facilitate the registration and recommendation of new therapies for CD.

## Conclusion

One century after the discovery by Chagas, progress has been made along the path to understanding and controlling CD; however, much remains to be done in order to truly be able to adequately treat this disease afflicting a reported 9.8 million patients [55]. The unmet medical needs of patients remain great, given the limitations of current drugs. Progress has been too little and too limited, with a small spectrum of chemical classes currently available as antitrypanosomal drugs or identified as druggable compounds. More activity and partnership is needed in order to **increase access to adequate and better-adapted diagnosis and treatment**.

Rooted in partnerships with all sectors and focused on patient needs, PDPs have shown that needs-driven innovation providing patients in resource-poor settings with important therapeutic improvements can be efficiently delivered, as seen with a number of improved malaria medicines [56]. As of 2004, 75% of active drug development projects for neglected diseases were conducted by PDPs, with eight to nine new drugs expected in the market by 2010 [57]. However, PDPs alone cannot meet the urgent needs of neglected patients.

Funding for R&D to improve treatments for CD is strikingly low, given the 100 million people at risk and CD's disease burden. Less than US$1 million (only 0.04% of R&D funding dedicated to neglected diseases) was spent on the development of new drugs for CD in 2007 [58]. For a disease extending its global fingers and for which no treatment exists for the chronic stage, the time to develop improved treatments is now. Through growing opportunities to act synergistically, public and private sectors must work together to make available a better treatment and tools for CD.

The increasing level of attention paid to CD in the new millennium offers reason for hope; greater efforts have been made to control CD, regional and worldwide research networks are being strengthened and built, and pharmaceutical companies have begun to share their libraries for neglected diseases. However, an opportunity was lost at this year's World Health Assembly when CD, at the 100th anniversary of its discovery, was dropped from the agenda due to concerns about a potential flu pandemic. A disease that continues to debilitate and kill people every day deserves to have more attention paid to it so that true innovation can be delivered to the patients in need of adequate treatments. Visit http://www.treatchagas.org to join the campaign.

## Supporting Information

Alternative Language Abstract S1 Translation of the abstract into Portuguese by Isabela Ribeiro
(0.01 MB PDF)

Alternative Language Abstract S2 Translation of the abstract into Spanish by Graciela Diap
(0.01 MB PDF)
