# Supplementary material for: New, Improved Treatments for Chagas Disease: From the R&D Pipeline to the Patients
Source: PLoS Negl Trop Dis. 2009 Jul 7;3(7):e484. doi: 10.1371/journal.pntd.0000484 (PMC2702098; doi:10.1371/journal.pntd.0000484)
Supplement: Alternative Language Abstract S1 — Translation of the abstract into Portuguese by Isabela Ribeiro (0.01 MB PDF) [file pntd.0000484.s001.pdf]

Ribeiro I, et al. New, improved treatments of Chagas disease: from the R&D pipeline to the patients.

Resumo em Português

Cem anos após a descoberta da doença de Chagas (DC), observam-se progressos no tratamento desta doença que ameaça um quarto da população da América Latina - com uma maior atenção dada recentemente a esta doença negligenciada, esforços como a "Iniciativa do Cone Sul" reduziram significativamente a transmissão da doença, assim como a decodificação do genoma do *Trypanosoma* em 2005. No entanto, no que diz respeito ao tratamento, **houve poucos e limitados progressos**. Dentre mais de 1.500 novos medicamentos desenvolvidos desde a década de 1960, apenas dois são para a DC. Nifurtimox e benznidazol possuem várias limitações: são direcionados à fase aguda da doença embora ainda não estejam disponíveis em concentrações pediátricas, apresentam toxicidade dose-dependente, eficácia limitada, longo período de tratamento e disponibilidade restrita. O desenvolvimento de novos e melhores tratamentos contra a DC é prioritário, pois a doença ameaça 100 milhões de pessoas na região endêmica e expande seu alcance com a globalização. Contudo, apenas 0,04% do financiamento (US$ 10 milhões) para P&D em doenças negligenciadas em 2007 foi atribuído para novos tratamentos contra a DC. A iniciativa Medicamentos para Doenças Negligenciadas, uma parceria para desenvolvimento de produtos sem fins lucrativos está trabalhando para desenvolver um portfólio robusto e bem equilibrado específico para DC. Sua prioridade é disponibilizar um tratamento eficaz, não tóxico e financeiramente acessível, com eficácia comprovada tanto na fase aguda como na crônica indeterminada. Outro trabalho em curso é o desenvolvimento de uma formulação de benznidazol com concentração pediátrica, o que poderia melhorar significativamente o manejo de pacientes. As mudanças observadas na última década oferecem um cenário favorável para colaboração e avanço na melhoria de tratamentos para doenças negligenciadas, tais como a DC. Porém, são necessários maiores investimentos (complementados por mecanismos de financiamento novos e adaptados) dos governos e do setor privado para assegurar que estes esforços sejam sólidos e sustentáveis.
