# Supplementary material for: New, Improved Treatments for Chagas Disease: From the R&D Pipeline to the Patients
Source: PLoS Negl Trop Dis. 2009 Jul 7;3(7):e484. doi: 10.1371/journal.pntd.0000484 (PMC2702098; doi:10.1371/journal.pntd.0000484)
Supplement: Alternative Language Abstract S2 — Translation of the abstract into Spanish by Graciela Diap (0.01 MB PDF) [file pntd.0000484.s002.pdf]

Ribeiro I, et al. New, improved treatments of Chagas disease: from the R&D pipeline to the patients.

Abstract in Spanish (author: Graciela Diap)

Cien años desde el descubrimiento de la enfermedad de Chagas (CD), se ha avanzado en el tratamiento de esta enfermedad que amenaza a una cuarta parte de la población que vive en América Latina - con una mayor atención recientemente otorgada a esta enfermedad olvidada, iniciativas como el INCOSUR-Chagas “Iniciativa del Cono Sur” han reducido considerablemente la transmisión de la enfermedad, así como la decodificación del genoma del *Trypanosoma* en 2005. Sin embargo, en lo que respecta a los tratamientos, los progresos han sido demasiado pocos y demasiado limitados. De los mas de 1.500 nuevos medicamentos desarrollados desde 1960, sólo 2 están disponibles para CD. Nifurtimox y benznidazol, tienen una serie de limitaciones: están dirigidos contra la fase aguda de la enfermedad, aún no están disponibles en formulaciones pediatricas, tienen toxicidad dosis-dependiente, una eficacia limitada, son tratamientos largos, y de una disponibilidad limitada. El desarrollo de nuevos y mejores tratamientos contra la CD es una prioridad para los 100 millones de personas a riesgo en regiones endémicas y para hacer frente a la expansión de la CD como consecuencia de la globalización; pero sólo el 0,04% ($10 millones) de fondos para I + D de enfermedades olvidadas se asignaron para nuevos tratamientos de CD en 2007. La Iniciativa Medicamentos para Enfermedades Olvidadas (DNDi), es una organización sin fines de lucro, dedicada a la investigación y el desarrollo de nuevos y mejores tratamientos para las enfermedades olvidadas que trabaja en la construcción de un portafolio especifico para CD, robusto y equilibrado, cuya prioridad es ofrecer un tratamiento eficaz, no tóxico y abordable de demostrada eficacia tanto en la fase aguda y crónica temprana de la CD. También se está trabajando en el desarrollo de una formulación pediátrica de benznidazol, que podría mejorar significativamente el manejo de los pacientes. Los cambios observados en la última década ofrecen un panorama favorable para colaborar y avanzar en la mejora de los tratamientos para las enfermedades olvidadas, como la CD, pero se necesitan mayores inversiones (complementadas con nuevos mecanismos de financiación adaptados) provenientes de gobiernos y del sector privado para garantizar que estos esfuerzos sean sólidos y sostenibles.
